# Breast cancer in women by HIV status: A report from the South African National Cancer Registry

**DOI:** 10.1371/journal.pone.0305274

**Published:** 2024-06-17

**Authors:** Maša Davidović, Tafadzwa Dhokotera, Isabel dos-Santos-Silva, Julia Bohlius, Mazvita Sengayi-Muchengeti

**Affiliations:** 1 Graduate School for Health Sciences, University of Bern, Bern, Switzerland; 2 Epidemiology and Public Health Department, Swiss Tropical and Public Health Institute, Allschwil, Switzerland; 3 University of Basel, Basel, Switzerland; 4 Graduate School for Cellular and Biomedical Sciences, University of Bern, Bern, Switzerland; 5 Department of Non-Communicable Disease Epidemiology, London School of Hygiene and Tropical Medicine, London, United Kingdom; 6 National Cancer Registry, National Health Laboratory Service, Johannesburg, South Africa; 7 School of Public Health, University of the Witwatersrand, Johannesburg, South Africa; University of Oxford, UNITED KINGDOM

## Abstract

**Background:**

Breast cancer (BC) is the leading cause of cancer-related morbidity and mortality in women living in South Africa, a country with a high HIV burden. However, characteristics of the double burden of HIV and BC in South Africa have not been properly investigated. We described characteristics of BC cases by HIV status in South Africa.

**Methods:**

In this nationwide South African study, we obtained BC records for women aged ≥15 years diagnosed in the public health sector between January 2004 and December 2014. We included records from the National Cancer Registry that had been linked to HIV-related laboratory records from the National Health Laboratory Service. We assessed the odds of being HIV positive versus HIV negative in relation to patient-, cancer-, and municipality-related characteristics.

**Results:**

From 2004–2014, 40 520 BC cases were diagnosed in women aged ≥15 years. Of these, 73.5% had unknown HIV status, 18.7% were HIV negative, and 7.7% were HIV positive. The median age at BC diagnosis was 43 years (interquartile range [IQR]: 37–52) in HIV positive and 57 years (IQR: 46–68) in HIV negative women, respectively. The odds of being HIV positive was higher for women who were aged 30–34 years compared to women aged 35–39 years at cancer diagnosis (odds ratio [OR] 1.38, 95% confidence interval [CI] 1.10–1.71), Black versus non-Black (OR 6.41, 95% CI 5.68–7.23), diagnosed with cancer in rural versus urban areas (OR 1.59, 95% CI 1.40–1.82) and diagnosed in municipalities with low and middle (OR 3.46, 95% CI 2.48–4.82) versus high socioeconomic position (OR 2.69, 95% CI 2.11–3.42).

**Conclusion:**

HIV status was unknown for the majority of BC patients. Among those with known HIV status, being HIV positive was associated with a younger age at cancer diagnosis, being Black and receiving care in municipalities of poor socioeconomic position. Future studies should examine opportunities to integrate HIV and BC control programs.

## Introduction

Breast cancer is the leading cause of cancer-related morbidity and mortality in women globally [1], and breast cancer incidence rates are rising in Sub-Saharan Africa [2]. The International Agency for Research on Cancer (IARC) estimated that in 2020, breast cancer accounted for 27.3% of all new cancer cases in women in sub-Saharan Africa [3], affecting more than 129 000 women in this region [4]. The rapid increase of breast cancer incidence rates in Sub-Saharan Africa are attributable to exogenous and endogenous factors. The risk factors associated with breast cancer are complex, and include changing population demographics and lifestyle, as well as environmental factors, genetics, and accessibility to screening and diagnostic services [1, 5–7]. Breast cancer is the most frequently diagnosed cancer among women in South Africa as well, accounting for 23% of all female cancers diagnosed in 2019 [8]. The rising burden of breast cancer in women in South Africa coincides with the high prevalence of HIV [3], where almost a quarter of women in their reproductive ages (15–49 years) are HIV positive (23.5%, CI 15.6–31.6) [9]. Over the past decade, governments in the African region, including South Africa, have made great political and economic efforts to fight HIV/AIDS and to increase access to antiretroviral therapy (ART), resulting in a significant increase of the average life expectancy of people living with HIV [4]. As a result, breast cancer cases are expected to be diagnosed more frequently in women living with HIV as well. In addition, existing challenges to timely detect, diagnose, and treat breast cancer patients in the region significantly influence the disease outcome, especially in groups of lower socio-economic position and women living with HIV [10].

In this South African nation-wide study, we described breast cancer cases in women aged 15 years and older diagnosed in public sector laboratories between 2004 and 2014. We evaluated the association between patient’s HIV status and age, ethnicity; tumour morphology and year of breast cancer diagnosis; urbanization and socio-economic position based on municipality of the cancer-reporting laboratory.

## Methods

### Study design and setting

The South African National Cancer Registry (NCR) has been conducting cancer surveillance since 1986 and serves as South Africa’s main source of national cancer incidence data. In this case-only study, we used records from the NCR to identify women diagnosed with breast cancer between January 2004 and December 2014. It is a pathology-based cancer registry, and both public and private laboratories are legislated to report all cancer cases to NCR [11]. The NCR is a division of the National Health Laboratory Services (NHLS), the largest diagnostic pathology service in the country. The NHLS provides laboratory and health services to over 80% of the national population through a network of public sector laboratories in all the nine provinces of South Africa [12]. The NHLS’ Corporate Data Warehouse (CDW) is a centralized electronic data repository for all public sector laboratory data, including HIV-related tests and cancer pathology reports.

### Study participants

From the South African NCR database, we retrieved all records of women aged 15 years and older (in this manuscript referred as “patients”) diagnosed with breast cancer in the public health sector from January 2004 to December 2014. We excluded cancer patient records from the private sector, since the source of HIV-related laboratory data used to determine the HIV status was provided by the NHLS’s CDW, which services the public sector only. We assumed that patients diagnosed with HIV in the public sector, would also have received a cancer diagnosis in the public sector, and that patients diagnosed with HIV in the private sector, would have received their cancer diagnosis in the private sector.

### Study variables, data source and measurement

We assessed patient-related characteristics: HIV status, age at breast cancer diagnosis, and ethnicity; cancer-related characteristics: tumour morphology and year of cancer diagnosis; and municipality-related characteristics: urbanization, municipality socio-economic position (SEP), and province. HIV status, age at breast cancer diagnosis and ethnicity were extracted from the cancer pathology records. For records where the HIV status was missing, the NCR used probabilistic record linkage methods to match HIV-related laboratory records from the NHLS’ CDW to determine HIV status. The HIV-related records included HIV diagnostic tests, CD4 cell counts and percentages, and HIV RNA viral loads from the public sector laboratories. Individuals were assigned HIV positive status if the pathology report indicated HIV positive status, if any HIV diagnostic test was positive or if HIV monitoring tests were recorded. HIV negative status was assigned if the HIV test results were negative. If the HIV result was indeterminate, unavailable or neither positive nor negative, the HIV status was considered unknown [13]. For cancer records where the information of the patient’s ethnicity was missing, the NCR used a hot-deck imputation method to impute missing ethnicity [14]. A reference database of approximately 1.4 million surnames with self-reported ethnicity was used to classify patients as Black, White, mixed ancestry and Indian/Asian, and unknown [14]. We used the International Classification of Diseases for Oncology (ICD-O-3) [15] coding system to identify breast cancer cases (topography code C50). We used the name of the cancer-reporting laboratory and determined its location (municipality) and SEP. The municipal SEP was based on the South African Index of Multiple Deprivation (the SAIMD) that was developed using census data [16]. The SAIMD describes multiple deprivation at ward level and combines indices of four domains or dimensions of deprivation (material, employment, education deprivation and living environment deprivation). The higher the SAIMD score, the more deprived the ward. The ward level SAIMD was then used to determine municipal SAIMD scores, by calculating the population weighted average rank of the wards within a municipality [16–18]. Patients were assigned the municipal SAIMD score based on the location of the laboratory that reported their breast cancer diagnosis. We also used the location of the laboratory providing breast cancer diagnosis to determine urbanization using the National Department of Health’s data dictionary [19].

### Data management

For analyses purposes, we classified age in 5-years age groups, and we combined the first two age groups into one (15–24 years) as the number of breast cancer cases was small. Tumour morphology was categorized based on ICD-O-3 classification (topography code C50) [15] as follows: ductal and lobular neoplasms, epithelial neoplasms, adenocarcinomas, and other morphology types of breast cancer. Ethnicity was defined as per Statistics South Africa groupings in Black, White, Coloured (mixed race), and Asian. However, because of an unequal distribution of case numbers among categories, we combined White, Coloured (mixed race) and Asian in non-Black group for statistical analyses. We categorized the year of breast cancer diagnosis for statistical analyses into the following categories: 2004–2006, 2007–2010, and 2011–2014. We defined the area where the cancer reporting facility was located as urban, rural, and unknown, according to the South African National Department of Health Data Dictionary [19]. We presented the municipality SEP in three categories based on the multiple deprivation rank of the municipality of the cancer reporting facility: low (≤78), middle (78–155) and high (>155). Provinces were defined according to the South African Government as Eastern Cape, Free State, Gauteng, KwaZulu-Natal, Limpopo, Mpumalanga, Northern Cape, North West, and Western Cape.

### Data analyses

For descriptive analyses, we presented results as frequencies and percentages for categorical variables, and mean and interquartile ranges [IQR] for continuous variables stratified by HIV status (positive, negative, and unknown). We conducted a Wilcoxon rank-sum test to compare median age at cancer diagnosis between patients who were HIV negative and HIV positive, and a chi-squared test to assess differences between patients with known and unknown HIV status; for both tests we set the a significance level at 0.05. In this case only study, we used univariable and multivariable logistic regression models to estimate odds ratios (ORs) with 95% confidence intervals (CIs) of being HIV positive versus HIV negative among breast cancer patients in relation to age at cancer diagnosis, ethnicity (Black and non-Black), year of cancer diagnosis, as well as municipality SEP and urbanization of cancer diagnosing facility. The logistic regression analyses were restricted to breast cancer cases with known HIV status. We conducted a sub-group analysis restricted to Black women diagnosed with breast cancer, as they comprised the majority of our dataset. We assessed interactions between HIV and other factors of interest (age, population group, and calendar period) using likelihood ratio tests at the 5% significance level. We used StataMP 16 (StataCorp Ltd, Texas, US) for all analyses.

### Ethical approval

We sought permission to use the routinely collected NHLS and NCR data from the Human Research Ethics Committee of the University of the Witwatersrand, Johannesburg, who possess appropriate ethical approvals for the Burden of Cancers Attributable to HIV in South Africa (2004–2014) (The BCAH)(Ethical Clearance Number: M160944) and for the South African HIV Cancer Match (SAM) Study (Protocol Ref No: M190594).

## Results

From 2004 to 2014, 664 870 cancer cases were reported to the South African NCR (S1 Fig). We excluded 335 589 records that were reported from the private sector. Of the 41 505 breast cancer cases reported, 978 cases were diagnosed in male patients and 7 cases in patients younger than 15 years. Finally, we included 40 520 breast cancer cases diagnosed in women aged 15 years and older in our study.

Table 1 presents patient-, cancer- and municipality-related characteristics by HIV status (positive, negative, unknown). The overall median age at breast cancer diagnosis was 55 years (IQR: 45–65 years). Ductal and lobular neoplasms were the most common morphology types, accounting for 85.2% of breast cancer cases. Most (60.1%) breast cancer patients were Black, 19.7% were White, 16.4% were Coloured, and 3.8% were Asian. Overall, most breast cancer cases were reported by laboratories located in urban areas (71.3%), in municipalities with high SEP (85.4%), and by public laboratories located in Gauteng (32.1%) and Western Cape provinces (26.2%).

**Table 1:** Characteristics of female breast cancer patients (n = 40 520) stratified by HIV status.

	HIV positive N (%)	HIV negative N (%)	HIV unknown N (%)	Total
**PATIENT-RELATED CHARACTERISTICS**				
**Age at cancer diagnosis [years]**				
15–24	27 (0.9)	29 (0.4)	131 (0.4)	187 (0.5)
25–29	127 (4.1)	114 (1.5)	422 (1.5)	663 (1.7)
30–34	381 (12.2)	280 (3.7)	1 004 (3.5)	1 665 (4.2)
35–39	544 (17.5)	563 (7.5)	1 793 (6.2)	2 900 (7.3)
40–44	591 (19.0)	822 (10.9)	2 772 (9.5)	4 185 (10.5)
45–49	499 (16.0)	1 029 (13.6)	3 380 (11.6)	4 908 (12.4)
50–54	374 (12.0)	1 079 (14.3)	3 460 (11.9)	4 913 (12.4)
55–59	258 (8.3)	988 (13.1)	3 486 (12)	4 732 (11.9)
60+	311 (10.0)	2 650 (35.1)	12 618 (43.4)	15 579 (39.2)
Missing	23 (n.a.)	37 (n.a.)	728 (n.a.)	788 (n.a.)
Median age (IQR)	43 (37–52)	54 (45–64)	57 (46–68)	55 (45–66)
**Ethnicity**				
Asian	29 (0.9)	187 (2.5)	1 277 (4.5)	1 493 (3.8)
Black	2 625 (86.2)	32 54 (44.1)	17 526 (61.4)	23 405 (60.1)
Colored	217 (7.1)	1 995 (27)	4 177 (14.6)	6 389 (16.4)
White	174 (5.7)	1 941 (26.3)	5 554 (19.5)	7 669 (19.7)
Missing	90 (n.a.)	214 (n.a.)	1 260 (n.a.)	1 564 (n.a.)
**CANCER-RELATED CHARACTERISTICS**				
**Tumour morphology**				
Ductal and Lobular Neoplasms	2 695 (86)	6 521 (85.9)	25 294 (84.9)	34 510 (85.2)
Epithelial Neoplasms, NOS	208 (6.6)	530 (6.7)	2 079 (6.7)	2 817 (6.9)
Adenocarcinomas	78 (2.5)	213 (2.8)	860 (2.9)	1 151 (2.8)
Others	154 (4.9)	327 (4.3)	1 561 (5.2)	2 042 (5.0)
**Year at cancer diagnosis**				
2004	70 (2.2)	114 (1.5)	2 915 (9.8)	3 099 (7.6)
2005	134 (4.3)	370 (4.9)	2 794 (9.4)	3 298 (8.1)
2006	167 (5.3)	450 (5.9)	2 872 (9.6)	3 489 (8.6)
2007	200 (6.4)	483 (6.4)	2 829 (9.5)	3 512 (8.7)
2008	282 (9.0)	575 (7.6)	2 886 (9.7)	3 743 (9.2)
2009	304 (9.7)	726 (9.6)	2 819 (9.5)	3 849 (9.5)
2010	354 (11.3)	763 (10.1)	2 827 (9.5)	3 944 (9.7)
2011	410 (13.1)	9 91 (13.1)	2 588 (8.7)	3 989 (9.8)
2012	452 (14.4)	1 076 (14.2)	2 776 (9.3)	4 304 (10.6)
2013	398 (12.7)	1 068 (14.1)	2 432 (8.2)	3 898 (9.6)
2014	364 (11.6)	975 (12.8)	2 056 (6.9)	3 395 (8.4)
**MUNICIPALITY- RELATED CHARACTERISTICS**				
**Urbanization**				
Rural	895 (29.6)	1 298 (17.1)	8 283 (32.0)	10 476 (28.7)
Urban	2 133 (70.4)	6 289 (82.9)	17 606 (68.0)	26 028 (71.3)
Missing	107 (n.a.)	4 (n.a.)	3 905 (n.a.)	4 016 (n.a.)
**Socio-economic position**				
Low	176 (5.8)	68 (0.9)	1 934 (7.5)	2 178 (6.0)
Middle	290 (9.6)	148 (2)	2 695 (10.4)	3 133 (8.6)
High	2 562 (84.6)	7 370 (97.2)	21 182 (82.1)	31 114 (85.4)
Missing	107 (n.a.)	5 (n.a.)	3 983 (n.a.)	4 095 (n.a.)
**Province**				
Gauteng	1 303 (43.0)	1 943 (25.6)	8 429 (32.7)	11 675 (32.1)
Western Cape	428 (14.1)	3 748 (49.4)	5 384 (20.9)	9 560 (26.2)
Eastern Cape	247 (8.2)	605 (8)	3 786 (14.7)	4 638 (12.7)
Free State	323 (10.7)	590 (7.8)	1 760 (6.8)	2 673 (7.3)
Limpopo	258 (8.5)	77 (1.0)	2 197 (8.5)	2 532 (7.0)
North West	216 (7.1)	290 (3.8)	1 415 (5.5)	1 921 (5.3)
Mpumalanga	149 (4.9)	62 (0.8)	1 229 (4.8)	1 440 (4.0)
Northern Cape	60 (2.0)	124 (1.6)	916 (3.6)	1 100 (3.0)
Kwazulu-Natal	44 (1.5)	148 (2.0)	695 (2.7)	887 (2.4)
Missing	107 (n.a.)	4 (n.a.)	3 983 (n.a.)	4 094 (n.a.)
**Total**	**3 135 (7.7)**	**7 591 (18.7)**	**29 794 (73.5)**	**40 520**

% = column percentages among women with no missing data, IQR = Interquartile range, n.a.–not applicable

**Fig 1:**
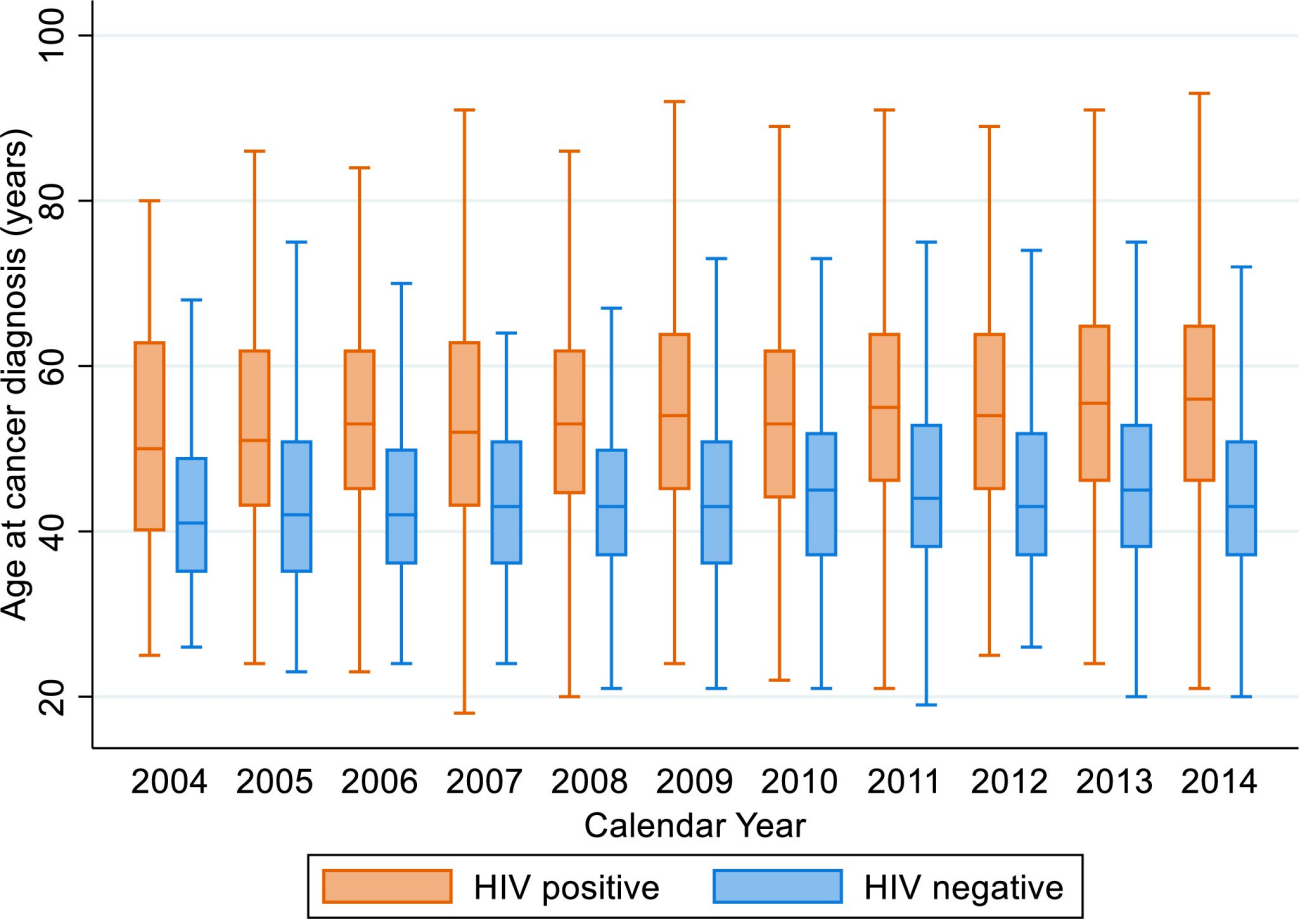
Median age at breast cancer diagnosis by HIV status and by year of cancer diagnosis.

Of all breast cancer patients, 7.7% were HIV positive, 18.7% were HIV negative, and in 73.5% the HIV status was unknown (Table 1). We found statistically significant differences in all patient-, cancer-, and municipality-related characteristics when comparing patients with known and unknown HIV status (S1 Table). The percentage of breast cancer patients with known HIV status (both HIV positive and negative) increased from 5.9% in 2004 to 39.4% in 2014. Among patients with known HIV status, the percentage of HIV positive declined from 38.0% in 2004 to 27.2% in 2014. Fig 1 shows median age at breast cancer diagnosis by HIV status and year of cancer diagnosis. The median age (IQR) was 43 years (37–52) for HIV positive, 54 years (45–64) for HIV negative and 57 years (46–68) for breast cancer patients with unknown HIV status. The difference in median of age at diagnosis between HIV positive and HIV negative breast cancer patients was statistically significant (p < 0.001) throughout the whole study period. Fifty-eight percent of the HIV positive breast cancer patients, but only 26.5% of the HIV negative and 23.3% of the HIV unknown, were younger than 45 years at the time of their breast cancer diagnosis. There were marked ethnic differences by HIV status with the percentage of women of Black ethnicity being much higher among HIV positive (86.2%) than among HIV negative patients (44.1%); the opposite was true for all the other ethnic groups. Tumour morphology did not vary by HIV status, with the large majority (85.2%) having a ductal or lobular morphology regardless of their HIV status. Most breast cancer patients were diagnosed in facilities that were located in an urban area (HIV positive 70.4%, HIV negative 82.9%, HIV status unknown 68.0%) and in municipalities with high SEP (HIV positive 84.6%, HIV negative 97.2%, HIV status unknown 82.1%). Forty-three percent of all HIV positive cases were diagnosed in laboratories located in Gauteng province, and 49.4% of all HIV negative cases were diagnosed in Western Cape Province.

**Fig 2:**
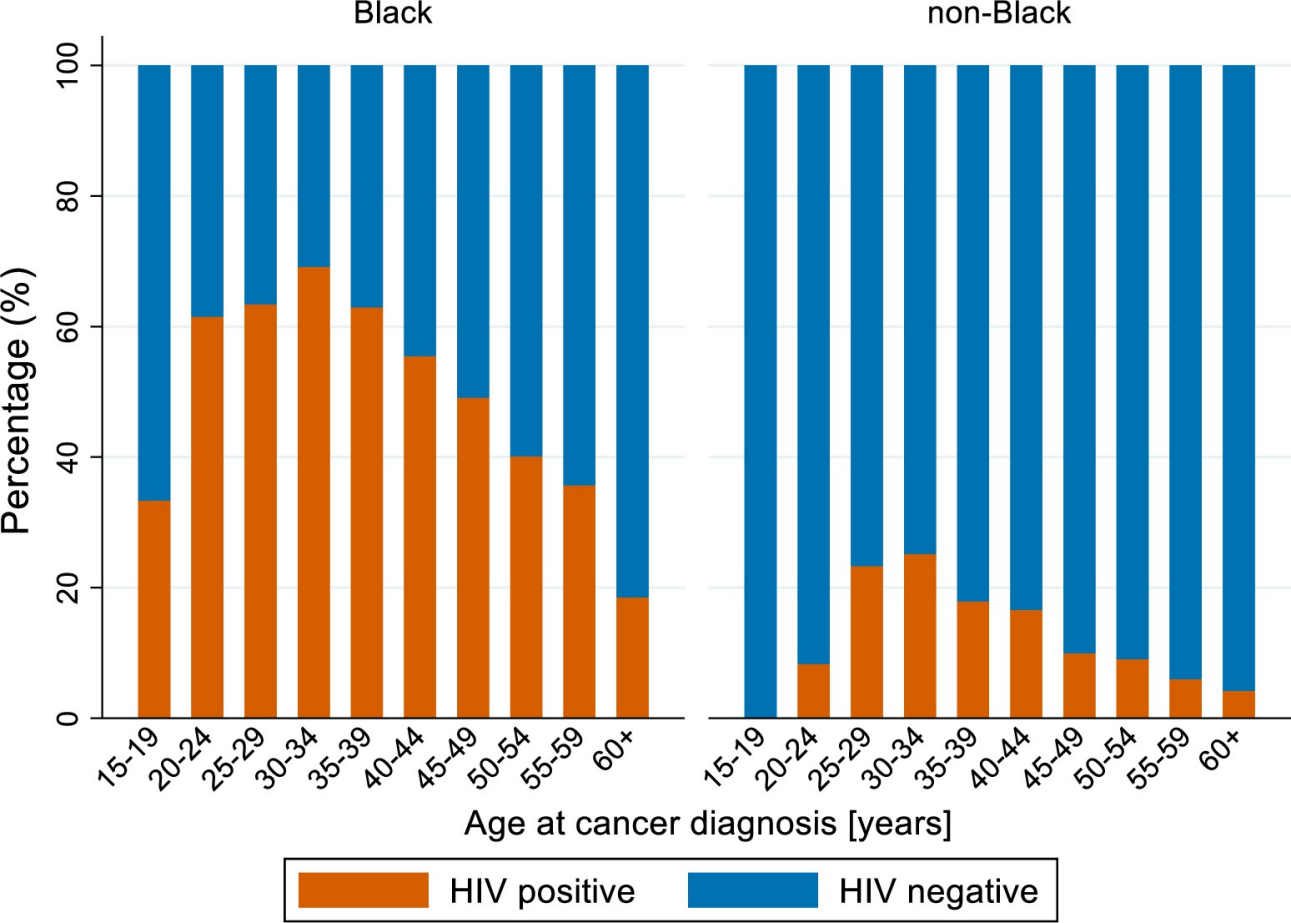
The percentage of black and non-black breast cancer patients with known HIV status by age at cancer diagnosis and HIV status (HIV positive and HIV negative).

**Fig 3:**
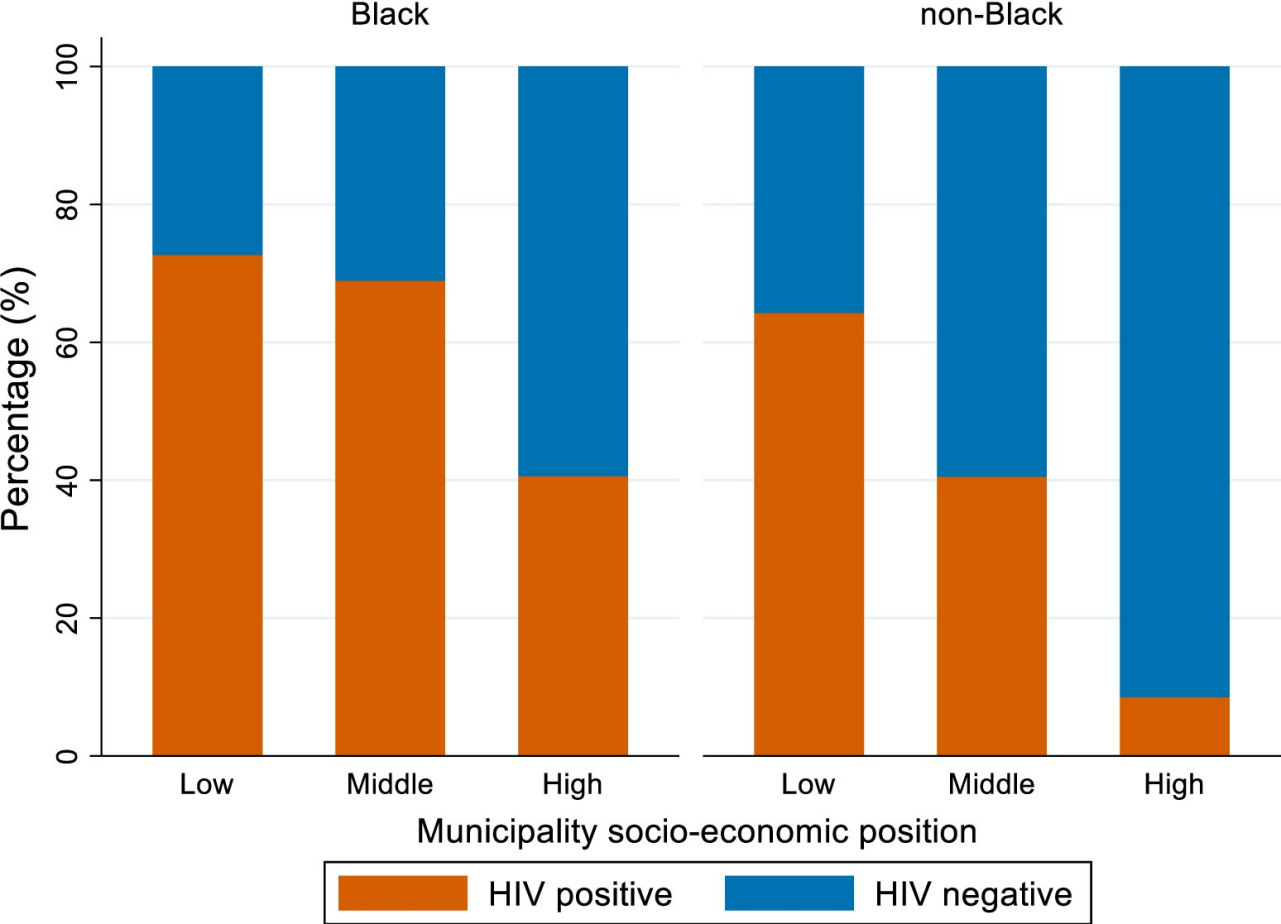
The percentage of black and non-black breast cancer patients with known HIV status by municipality socio-economic position (SEP) and HIV status (HIV positive and HIV negative).

The percentage of breast cancer cases by age and HIV status differed between Black and non-Black patients. In breast cancer patients with known HIV status (Fig 2), there were more HIV positive than HIV negative cases in each age group younger than 45 years in Black patients. In non-Black patients, there were more HIV negative than HIV positive breast cancer patients in each age category. The highest percentage of both Black and non-Black breast cancer patients were diagnosed in laboratories located in municipalities with high SEP (Table 1). Among breast cancer patients with known HIV status (Fig 3), a higher percentage of HIV positive patients were diagnosed in laboratories located in municipalities with low and middle SEP compared to HIV negative patients. This was more prominent among Black patients.

**Fig 4:**
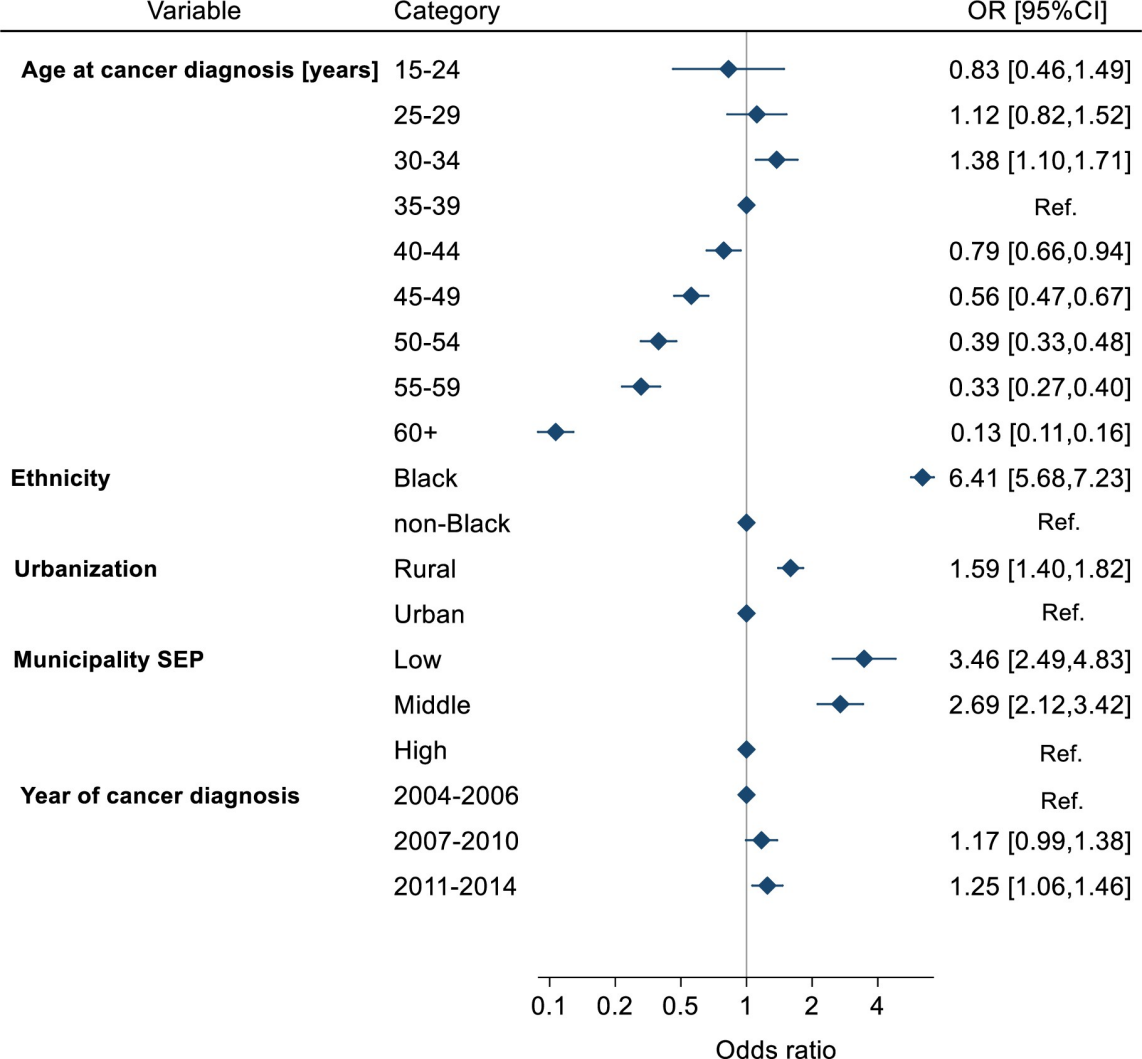
Multivariable logistic regression: Factors associated with being HIV positive in breast cancer female patients. Factors included in the model: age at cancer diagnosis, ethnicity, year of cancer diagnosis, municipality socio-economic position (SEP) and urbanization. CI–confidence interval; OR–odds ratio; Ref.–reference category.

Results from univariable and multivariable logistic regressions were similar, see S2 Table. In the complete-case multivariable logistic regression model (Fig 4), patients who were diagnosed with breast cancer at ages 30–34 years were more likely to be HIV positive compared to women aged 35–39 years (OR 1.38, 95% CI 1.10–1.71). The odds of being HIV positive decreased progressively for ages above 39 years. The odds of being HIV positive was about six times (OR 6.41, 95% CI 5.68–7.23) higher among Black breast cancer patients compared to non-Black women. Breast cancer patients who were diagnosed in laboratories located in rural areas were 1.6 times more likely to be HIV positive compared to breast cancer patients who were diagnosed in laboratories located in urban areas (OR 1.59, 95% CI 1.40–1.82). Patients whose breast cancer was diagnosed in public laboratories located in municipalities with low and middle SEP were more likely to be HIV positive compared to patients diagnosed in municipalities with high SEP (OR 3.46, 95% CI 2.48–4.82 and OR 2.69, 95% CI 2.11–3.42, respectively). The odds of being HIV positive increased gradually over time, being highest among patients who were diagnosed with breast cancer in the most recent study years, i.e., 2011–2014 (OR 1.25, 95% CI 1.06–1.46). In a sub-group analysis restricted to Black women, our findings were confirmed (S3 Table).

## Discussion

In this South-African nation-wide study, we evaluated 40 520 breast cancer cases in women aged 15 years and older, diagnosed in a public health sector laboratory between 2004 and 2014. Our study has shown that the median age at breast cancer diagnosis was 10 years lower in breast cancer patients who were HIV positive as compared to breast cancer patients who were HIV negative. Black breast cancer patients were almost seven times more likely to be diagnosed with HIV compared to non-Black breast cancer patients.

Our study had several limitations. The South African NCR is a pathology-based registry, and cancer cases that are diagnosed radiologically and clinically only are not captured. This may lead to underreporting of breast cancer cases. We lacked clinical (i.e., stage and receptor status of breast cancer at diagnosis) and patient information that is associated with the development of breast cancer, such as tobacco use, alcohol consumption, overweight and obesity, lack of physical activity, older age at first birth, low fertility, or family history. In our study population, HIV status was unknown for most patients. A high percentage of missing data for HIV status potentially introduced selection bias and limited the generalizability of our findings. We could not compare the risk of developing breast cancer in HIV positive versus HIV negative women, as our dataset included breast cancer cases only. To describe municipality SEP, we used small area level estimates of deprivation generated from national consensus ward-level data [16–18]. This approach assumed a uniform level of deprivation areas across the municipality, which not necessarily reflect the actual distribution of deprivation. While it is designed to provide highly accurate estimates, there is a risk of ecological bias if not interpreted correctly.

The relationship between HIV and breast cancer in women is not fully understood [20]. While one review from South Africa found an increased risk of breast cancer in HIV positive women [21], other African studies did either not find evidence for an association [22–24], or they found a positive association between HIV and breast cancer [25]. In our study, 29.2% of breast cancer patients with a known HIV status were HIV positive. South African hospital-based studies of women diagnosed with breast cancer conducted between 2006 and 2014, reported that 18–20% patients were HIV positive [22, 26, 27]. Another prospective study that was examining women newly diagnosed with breast cancer in six public hospitals in South Africa, reported that 22% of breast cancer patients were HIV positive [28]. While the prevalence of HIV positive breast cancer patients in our cohort seems high, it is important to note that in cases where HIV status was missing, text mining of pathology reports was used to obtain HIV status. Doctors are more likely to note down the HIV status of an HIV positive patient increasing the likelihood of picking up those that were tested positive compared to those that tested negative or never tested.

The median age at breast cancer diagnosis in our study (55 years) was similar to that reported in previous studies that included women from several African countries, including South Africa [27, 29–33]. In Sub-Saharan Africa, patients with breast cancer generally present at a relatively early age regardless of HIV status [20, 34]. The different age distributions of the underlying population can mainly explain the apparently early onset breast cancer in African women [34, 35]. In African countries, the population structure is skewed towards younger age groups, and the number of new breast cancer cases are expected to be higher where the female population is larger. Therefore, lower median age at breast cancer diagnosis does not necessarily mean that younger women are more likely to have breast cancer [34, 35]. Likewise, in our study HIV positive patients were diagnosed with breast cancer on average 10 years earlier than HIV negative patients were. This is in line with previous studies evaluating HIV positive breast cancer patients in South Africa [22, 27, 28, 32, 33, 36]. The age difference at breast cancer diagnosis between HIV positive and HIV negative patients has been discussed in previous studies. As the HIV positive population is on average younger than the HIV negative population [37] this will likely explain the apparent differences and may not carry an etiological significance.

Among breast cancer patients with known HIV status, the HIV prevalence in Black patients was 44.6% as compared to less than 10% in non-Black patients. Other studies that assessed HIV status in South African breast cancer patients also have found that HIV prevalence in Black breast cancer patients was higher compared to the HIV prevalence in non-Black patients; and it ranged from 18.4% to 33.7% [22, 26–28, 36, 38–40]. We assumed that in our study based on routine care data, healthcare providers might have offered HIV tests more often to Black patients than to non-Black patients, as Black people in South Africa are at higher risk for contracting HIV as compared to non-Black people [41]. Nevertheless, the reasons for the higher HIV prevalence in Black breast cancer patients in our study, in comparison to other studies, remain unclear. The percentage of breast cancer patients with unknown HIV decreased from 94.1% in 2004 to 60.6% in 2014. This might reflect improving HIV testing services availability in South Africa across the study period and is in line with previous studies in South Africa [22]. Still, many women diagnosed with breast cancer were unaware of their HIV status by the end of our study period, especially in women aged older than 50 years. This is supported by an earlier study on HIV testing patterns in South Africa, that found that older cancer patients were less likely to be tested for HIV than younger patients [39]. They also reported that 14.3% HIV positive patients diagnosed with breast cancer were unaware of their HIV status.

In our study, most patients, regardless of HIV status, were diagnosed with breast cancer in laboratories located in urban municipalities or in municipalities with high SEP. Our measures of SEP and urbanization describe the municipality where the breast cancer diagnosis laboratory is located, it does not describe a woman’s residential or her individual socio-economic circumstances. Nevertheless, it is well-established that area socio-economic factors affect the risk of developing breast cancer as well as the likelihood of the disease being diagnosed at an early stage and properly managed. A recent IARC study [42] found that incidence rates of breast cancer are increasing with increasing levels of socioeconomic development. This can be explained by the higher exposure to relevant risk factors, such as tobacco use, alcohol consumption, overweight and obesity, lack of physical activity, low fertility, and older age at first birth, shorter duration of breastfeeding and later age at menopause, as countries are progressing from low to very high socioeconomic development. A South African study of breast cancer in black woman found that higher household socioeconomic status reduced the odds of having advanced-stage breast cancer at diagnosis [31]. Another South African study explored female specific cancers and their risk factors in women living with HIV and reported that diagnosis of breast cancer was strongly associated with municipalities with high SEP [43]. South African women who were relatively wealthier, better educated [38], with higher socioeconomic status [31], or who are living close to health facility or hospital [26] were more likely to be diagnosed with breast cancer earlier. Hence, women from communities with low SEP and in rural areas are likely to have delayed breast cancer diagnosis. We also found that breast cancer patients diagnosed in laboratories in rural municipalities with low or middle SEP are more likely to be diagnosed with HIV compared to their counterparts. In South Africa, socioeconomic factors such as unstable housing and lower education level impact the odds of HIV infection [44].

Future research should aim to properly address and quantify the anticipated increasing numbers of women with breast cancer and HIV in South Africa as well as in other African settings with a high HIV prevalence. More research is needed to understand HIV testing practices in healthcare facilities, and breast cancer patients should be offered HIV testing if they do not know their status. Integrating breast cancer detection programs into existing health services, e.g., HIV/AIDS clinics should be considered, but with caution to not overload existing infrastructure and workforce. In the resource-limited settings, down staging of the disease may be achieved by improving awareness among women, communities, and health professionals [32, 45, 46], in addition to supporting early detection and improving timely access to appropriate treatment [47]. Addressing structural, sociocultural, personal and financial barriers to early presentation and diagnosis, and sustainable community and healthcare worker education may reduce breast cancer morbidity and mortality [30, 47–49]. Although the association between HIV and breast cancer is still unclear, it is evident that HIV low socio-economic circumstances are associated with poor survival from breast cancer [20, 32, 36, 37, 50]. Novel approaches to manage and treat multiple comorbidities in African women are needed. To quantify the double burden from breast cancer and HIV infection, high quality population-based data are essential. Improvements in cancer data collection and linkage of clinical and mortality data to existing cancer surveillance programs are urgently needed. In addition, socioeconomic data on individual level, as opposed to area-based level, can enhance insights into the social and economic impact on the risk of developing of breast cancer and on its outcomes. By exploring both perspectives, we can achieve a more comprehensive understanding of these factors.

## Conclusion

Our study provides insights on breast cancer cases stratified by HIV status and contributes to better understand the double burden from HIV and breast cancer in South Africa. With the ageing of the population living with HIV, we can expect a growing burden of breast cancer in this population, which may have a significant impact on the health and quality of life of women living with HIV. Integrating breast cancer prevention and diagnostic services in well-established HIV clinics may be a first step toward addressing global disparities in access to, and availability of, breast cancer detection. In South Africa, as well as in other countries with high HIV burden, newly diagnosed breast cancer patients who are unaware of their HIV status should be offered HIV testing to ensure patients are properly managed.

## Supporting information

S1 Fig Flow chart of study cases selection. (PDF)

S1 File Minimum data set. (XLSX)

S1 Table Characteristics of female breast cancer patients (n = 40 520) stratified by HIV status (known, unknown). (PDF)

S2 Table Univariable and multivariable analysis for different explanatory variables in HIV positive breast cancer patients compared to HIV negative breast cancer patients. (PDF)

S3 Table Sub-group analysis–univariable and multivariable analysis for different explanatory variables in HIV positive black breast cancer patients compared to HIV negative black breast cancer patients. (PDF)

## Abbreviations

ART: antiretroviral therapy
CDW: Corporate Data Warehouse
CIs: Confidence Intervals
HIV: Human Immunodeficiency Virus
IARC: The International Agency for Research on Cancer
ICD-O-3: The International Classification of Diseases for Oncology
IQR: Interquartile range
NCR: National Cancer Registry
NHLS: The National Health Laboratory Services
ORs: Odds Ratios
RNA: Ribonucleic Acid
SEP: The Socio-Economic Position
The SAIMD 2011: The South African Multiple Deprivation Index for 2011
