# Supplementary material for: Breast cancer in women by HIV status: A report from the South African National Cancer Registry
Source: PLoS One. 2024 Jun 17;19(6):e0305274. doi: 10.1371/journal.pone.0305274 (PMC11182510; doi:10.1371/journal.pone.0305274)

Supporting information

S1 Figure. Flow chart selection of study cases from the South African National Cancer Registry in study period (2004-2014)

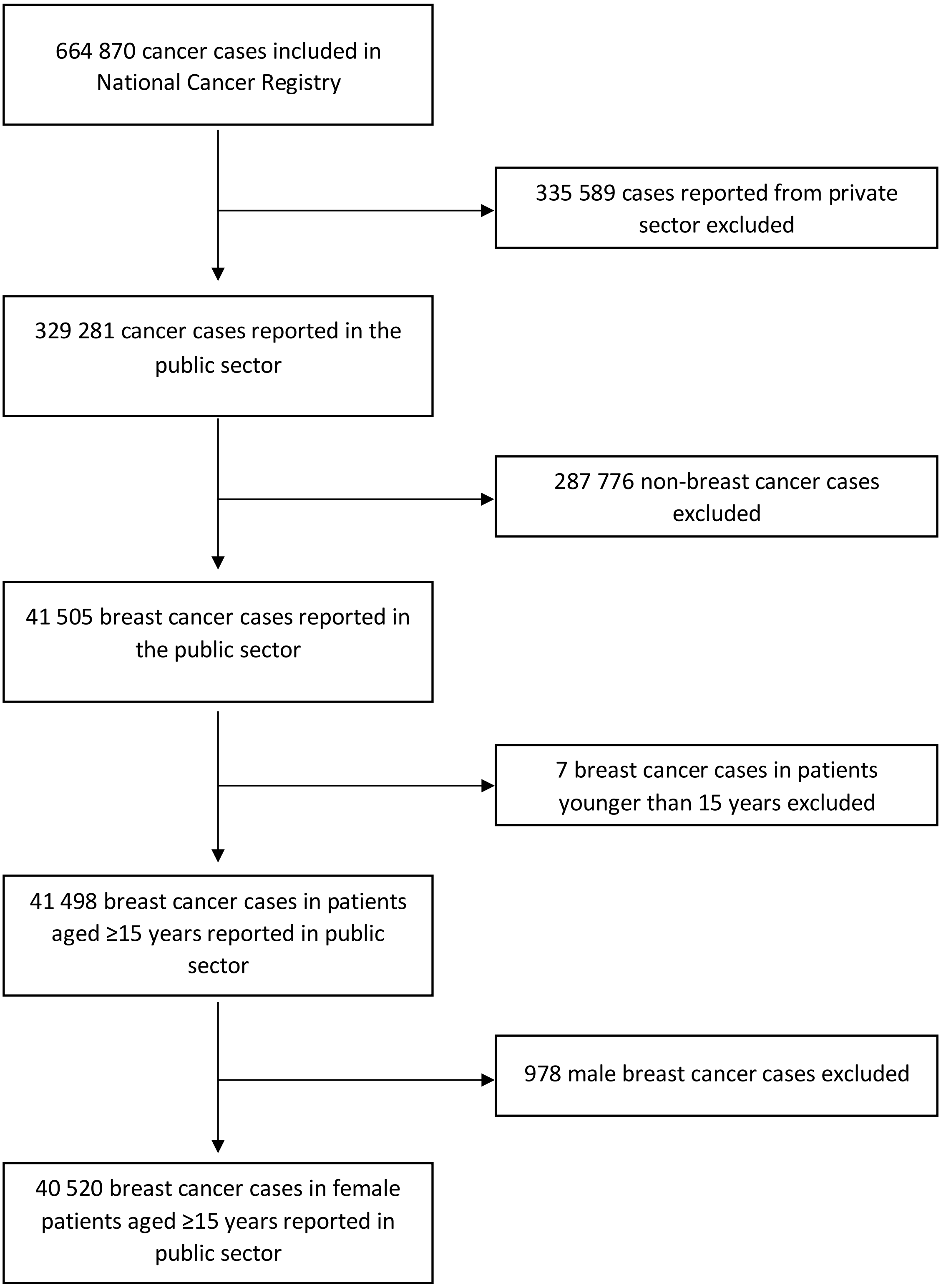

Supplement: S1 Fig — (PDF) [file pone.0305274.s001.pdf]
