# Supplementary material for: Breast cancer in women by HIV status: A report from the South African National Cancer Registry
Source: PLoS One. 2024 Jun 17;19(6):e0305274. doi: 10.1371/journal.pone.0305274 (PMC11182510; doi:10.1371/journal.pone.0305274)
Supplement: S1 Table — (PDF) [file pone.0305274.s003.pdf]

Supporting information

S1 Table. Characteristics of female breast cancer patients (n=40 520) stratified by HIV status (known, unknown)

	HIV known N (%)	HIV unknown N (%)	Total N (%)	P-value
**Patient-level characteristics**				
**Age at cancer diagnosis [years]**				
15-24	56 (0.5)	131 (0.4)	187 (0.5)	
25-29	241 (2.3)	422 (1.5)	663 (1.7)	
30-34	661 (6.2)	1 004 (3.5)	1 665 (4.2)	
35-39	1 107 (10.4)	1 793 (6.2)	2 900 (7.3)	
40-44	1 413 (13.3)	2 772 (9.5)	4 185 (10.5)	
45-49	1 528 (14.3)	3 380 (11.6)	4 908 (12.4)	< 0.001
50-54	1 453 (13.6)	3 460 (11.9)	4 913 (12.4)	
55-59	1 246 (11.7)	3 486 (12)	4 732 (11.9)	
60+	2 961 (27.8)	12 618 (43.4)	15 579 (39.2)	
Missing	60 (n.a.)	728 (n.a.)	788 (n.a.)	
Median age (IQR)	51 (42 – 61)	57 (46 – 68)	55 (45-66)	
**Ethnicity**				
Asian	216 (2.1)	1 277 (4.5)	1 493 (3.8)	
Black	5 879 (56.4)	17 526 (61.4)	23 405 (60.1)	
Colored	2 212 (21.2)	4 177 (14.6)	6 389 (16.4)	< 0.001
White	2 115 (20.3)	5 554 (19.5)	7 669 (19.7)	
Missing	304 (n.a.)	1 260 (n.a.)	1 564 (n.a.)	
**Cancer-level characteristics**				
**Tumour morphology**				
Ductal and Lobular Neoplasms	9 216 (85.9)	25 294 (84.9)	34 510 (85.2)	
Epithelial Neoplasms, NOS	738 (6.9)	2 079 (6.7)	2 817 (6.9)	= 0.01
Adenocarcinomas	291 (2.7)	860 (2.9)	1 151 (2.8)	
Others	481 (4.5)	1 561 (5.2)	2 042 (5.0)	
**Year at cancer diagnosis**				
2004	184 (1.7)	2 915 (9.8)	3 099 (7.7)	
2005	504 (4.7)	2 794 (9.4)	3 298 (8.1)	
2006	617 (5.8)	2 872 (9.6)	3 489 (8.6)	
2007	683 (6.4)	2 829 (9.5)	3 512 (8.7)	
2008	857 (8.0)	2 886 (9.7)	3 743 (9.2)	
2009	1 030 (9.6)	2 819 (9.5)	3 849 (9.5)	< 0.001
2010	1 117 (10.4)	2 827 (9.5)	3 944 (9.7)	
2011	1 401 (13.1)	2 588 (8.7)	3 989 (9.8)	
2012	1 528 (14.3)	2 776 (9.3)	4 304 (10.6)	
2013	1 466 (13.7)	2 432 (8.2)	3 898 (9.6)	
2014	1 339 (12.5)	2 056 (6.9)	3 395 (8.4)	

**Municipality-level characteristics**				
**Urbanization**				
Rural	2 193 (20.7)	8 283 (32.0)	10 476 (28.7)	
Urban	8 422 (79.3)	17 606 (68.0)	26 028 (71.3)	< 0.001
Missing	111 (n.a.)	3 905 (n.a.)	4 016 (n.a.)	
**Socio-economic position**				
Low	244 (2.3)	1 934 (7.5)	2 178 (6.0)	
Middle	438 (4.1)	2 695 (10.4)	3 133 (8.6)	
High	9 932 (93.6)	21 182 (82.1)	31 114 (85.4)	< 0.001
Missing	112 (n.a.)	3 983 (n.a.)	4 095 (n.a.)	
**Province**				
Gauteng	3 246 (30.6)	8 429 (32.7)	11 675 (32.1)	
Western Cape	4 176 (39.3)	5 384 (20.9)	9 560 (26.2)	
Eastern Cape	852 (8.0)	3 786 (14.7)	4 638 (12.7)	
Free State	913 (8.6)	1 760 (6.8)	2 673 (7.3)	
Limpopo	335 (3.2)	2 197 (8.5)	2 532 (7.0)	
North West	506 (4.8)	1 415 (5.5)	1 921 (5.3)	< 0.001
Mpumalanga	211 (2.0)	1 229 (4.8)	1 440 (4.0)	
Northern Cape	184 (1.7)	916 (3.6)	1 100 (3.0)	
Kwazulu-Natal	192 (1.8)	695 (2.7)	887 (2.4)	
Missing	111 (n.a.)	3 983 (n.a.)	4 094 (n.a.)	
**Total**	**10 726 (26.5)**	**29 794 (73.5)**	**40 520**	
