# Supplementary material for: Breast cancer in women by HIV status: A report from the South African National Cancer Registry
Source: PLoS One. 2024 Jun 17;19(6):e0305274. doi: 10.1371/journal.pone.0305274 (PMC11182510; doi:10.1371/journal.pone.0305274)
Supplement: S2 Table — (PDF) [file pone.0305274.s004.pdf]

Supporting information

S2 Table. Univariable and multivariable analysis for different explanatory variables in HIV positive breast cancer patients compared to HIV negative breast cancer patients

	**Univariable analyses OR (95% CI)**	**Multivariable analyses OR (95% CI)**
**Patient-level characteristics**		n=10 258
**Age at cancer diagnosis [years]**		
15-24	0.96 (0.56-1.65)	0.83 (0.46-1.49)
25-29	1.15 (0.87-1.52)	1.12 (0.82-1.52)
30-34	1.41 (1.16-1.71)	1.38 (1.10-1.71)
35-39	Ref.	Ref.
40-44	0.74 (0.64-0.87)	0.79 (0.66-0.94)
45-49	0.50 (0.43-0.59)	0.56 (0.47-0.67)
50-54	0.36 (0.30-0.42)	0.39 (0.33-0.48)
55-59	0.27 (0.23-0.32)	0.33 (0.27-0.40)
60+	0.12 (0.10-0.14)	0.13 (0.11-0.16)
**Ethnicity**		
Black	7.92 (7.07-8.86)	6.41 (5.68-7.23)
Non-Black	Ref.	Ref.
**Year of cancer diagnosis**		
2004-2006	Ref.	Ref.
2007-2010	1.13 (0.98-1.29)	1.17 (0.99-1.38)
2011-2014	0.99 (0.87-1.14)	1.25 (1.06-1.46)
**Municipality-level characteristics**		
**Urbanization**		
Rural	2.03 (1.84-2.24)	1.59 (1.40-1.82)
Urban	Ref.	Ref.
**Socio-economic position**		
Low	7.45 (5.61-9.89)	3.46 (2.48-4.82)
Middle	5.64 (4.60-6.91)	2.69 (2.11-3.42)
High	Ref.	Ref.

CI: confidence interval; n – number of observations: OR – odds ratio; Ref. – reference group.
