# Supplementary material for: Breast cancer in women by HIV status: A report from the South African National Cancer Registry
Source: PLoS One. 2024 Jun 17;19(6):e0305274. doi: 10.1371/journal.pone.0305274 (PMC11182510; doi:10.1371/journal.pone.0305274)
Supplement: S3 Table — (PDF) [file pone.0305274.s005.pdf]

Supporting information

S3 Table. Sub-group analysis – univariable and multivariable analysis for different explanatory variables in HIV positive Black breast cancer patients compared to HIV negative Black breast cancer patients

	Univariable analyses OR (95% CI)	Multivariable analyses OR (95% CI)
**Patient-level characteristics**		n=5 739
**Age at cancer diagnosis [years]**		
15-24	0.86 (0.46-1.63)	0.93 (0.49-1.78)
25-29	1.02 (0.72-1.44)	1.07 (0.75-1.51)
30-34	1.32 (1.03-1.68)	1.32 (1.03-1.7)
35-39	Ref.	Ref.
40-44	0.73 (0.60-0.89)	0.73 (0.60-0.90)
45-49	0.57 (0.46-0.69)	0.57 (0.46-0.70)
50-54	0.39 (0.32-0.48)	0.38 (0.30-0.47)
55-59	0.33 (0.26-0.41)	0.33 (0.26-0.42)
60+	0.13 (0.11-0.16)	0.12 (0.10-0.15)
**Year of cancer diagnosis**		
2004-2006	Ref.	Ref.
2007-2010	1.03 (0.87-1.22)	1.14 (0.95-1.37)
2011-2014	1.20 (1.02-1.41)	1.35 (1.12-1.61)
**Municipality-level characteristics**		
**Residence**		
Rural	1.95 (1.72-2.20)	1.62 (1.38-1.89)
Urban	Ref.	Ref.
**Socio-economic position**		
Low	3.89 (2.89-5.24)	3.05 (2.16-4.31)
Middle	3.25 (2.60-4.05)	2.50 (1.94-3.22)
High	Ref.	Ref.

CI – confidence interval; n – number of observations; OR – odds ratio; Ref. – reference group.
